# Exclusion of the Possibility of “False Ripples” From Ripple Band High-Frequency Oscillations Recorded From Scalp Electroencephalogram in Children With Epilepsy

**DOI:** 10.3389/fnhum.2021.696882

**Published:** 2021-06-15

**Authors:** Katsuhiro Kobayashi, Takashi Shibata, Hiroki Tsuchiya, Tomoyuki Akiyama

**Affiliations:** Department of Child Neurology, Okayama University Graduate School of Medicine, Dentistry and Pharmaceutical Sciences and Okayama University Hospital, Okayama, Japan

**Keywords:** epilepsy, child, scalp EEG, false ripple, high-frequency oscillation (HFO), fast oscillation (FO)

## Abstract

**Aim:**

Ripple-band epileptic high-frequency oscillations (HFOs) can be recorded by scalp electroencephalography (EEG), and tend to be associated with epileptic spikes. However, there is a concern that the filtration of steep waveforms such as spikes may cause spurious oscillations or “false ripples.” We excluded such possibility from at least some ripples by EEG differentiation, which, in theory, enhances high-frequency signals and does not generate spurious oscillations or ringing.

**Methods:**

The subjects were 50 pediatric patients, and ten consecutive spikes during sleep were selected for each patient. Five hundred spike data segments were initially reviewed by two experienced electroencephalographers using consensus to identify the presence or absence of ripples in the ordinary filtered EEG and an associated spectral blob in time-frequency analysis (Session A). These EEG data were subjected to numerical differentiation (the second derivative was denoted as EEG″). The EEG″ trace of each spike data segment was shown to two other electroencephalographers who judged independently whether there were clear ripple oscillations or uncertain ripple oscillations or an absence of oscillations (Session B).

**Results:**

In Session A, ripples were identified in 57 spike data segments (Group A-R), but not in the other 443 data segments (Group A-N). In Session B, both reviewers identified clear ripples (strict criterion) in 11 spike data segments, all of which were in Group A-R (*p* < 0.0001 by Fisher’s exact test). When the extended criterion that included clear and/or uncertain ripples was used in Session B, both reviewers identified 25 spike data segments that fulfilled the criterion: 24 of these were in Group A-R (*p* < 0.0001).

**Discussion:**

We have demonstrated that real ripples over scalp spikes exist in a certain proportion of patients. Ripples that were visualized consistently using both ordinary filters and the EEG″ method should be true, but failure to clarify ripples using the EEG″ method does not mean that true ripples are absent.

**Conclusion:**

The numerical differentiation of EEG data provides convincing evidence that HFOs were detected in terms of the presence of such unusually fast oscillations over the scalp and the importance of this electrophysiological phenomenon.

## Introduction

High-frequency oscillations (HFOs) have attracted attention due to their close relationship with epileptogenicity (Jacobs et al., 2010; Akiyama et al., 2011; Frauscher et al., 2017; Thomschewski et al., 2019). Ripple band epileptic HFOs can be recorded from a scalp electroencephalogram (EEG; Kobayashi et al., 2010; Andrade-Valenca et al., 2011; Zelmann et al., 2014; von Ellenrieder et al., 2014; Shibata et al., 2016; Bernardo et al., 2018), and they are suggested to indicate disease severity particularly in children with developmental and epileptic encephalopathy, such as West syndrome (Kobayashi et al., 2011; Kobayashi et al., 2015; van Klink et al., 2016; Nariai et al., 2020). Epileptic ripples, however, tend to be associated with epileptic discharges or spikes, and there is a concern that filtration of steep waveforms such as spikes may cause spurious oscillations or “false ripples” (Bénar et al., 2010). Although time–frequency analysis (TFA) supplements HFO detection, TFA is not a perfect solution because the spectra are based on frequency analysis, and occasionally, noisy and high-frequency spectral blobs may be buried in lower-frequency activities.

We aimed to exclude the possibility of such false ripples from at least some ripples that were observed in association with scalp spikes using numerical differentiation processing of EEG data, which relatively enhances high-frequency signals and does not generate spurious oscillations or ringing, in theory. There are several methods to reduce slow frequency activity (i.e., whitening) to improve HFO detectability, but avoiding the generation of spurious oscillations has not received much attention (Roehri et al., 2016). In the present study, we intended to show that ripples really exist in association with spikes using a straightforward method.

## Materials and Methods

### Background

The derivative of sine waves with a certain frequency yields sine waves that have the identical frequency and a phase shift. Let *x* denote a sine function of time (*t*) in the original data:

x=A⋅sin⁡(2πft)

where *A* and *f* denote amplitude and frequency, respectively.

Its first derivative is indicated as:

$$x^{\prime }=2\pi fA\cdot \cos\left(2\pi ft\right)=2\pi fA\cdot \sin\left(2\pi ft+\frac{\pi }{2}\right).$$

It indicates the degree of temporal EEG change (denoted herein as EEG’ according to the prime notation of derivative). Let *X*(*t*) denote EEG potential data at time point *t*, the numerical approximation of EEG’ is defined simply as the potential difference between two adjacent time points divided by the sampling interval (Δ*t*), which was 2 ms in the present study with the sampling rate at 500 Hz, as follows:

$$X^{\prime }\left(t\right)=\left\{X\left(t+\Delta t\right)-X\left(t\right)\right\}/\Delta t.$$

The second derivative is denoted as EEG″, and it is obtained by differentiation of the first derivative, as follows:

$$x^{{}^{\prime \prime }}=-4\pi^{2}f^{2}A\cdot \sin\left(2\pi ft\right)=4\pi^{2}f^{2}A\cdot \sin\left(2\pi ft+\pi \right).$$

Its numerical approximation is indicated as follows:

$$\begin{array}{c} X^{\prime \prime }\left(t\right)=\frac{\left\{X^{\prime }\left(t+\Delta t\right)-X^{\prime }\left(t\right)\right\}}{\Delta t}=\\ \left\{X\left(t+2\Delta t\right)-2X\left(t+\Delta t\right)+X\left(t\right)\right\}/\Delta t^{2}. \end{array}$$

This equation denotes a type of finite impulse response (FIR) filter that has only three coefficients, which limit the responses within the duration of these samples (3Δ*t* = 6 ms) and, therefore, do not allow ringing or the generation of spurious oscillations with ≥4 cycles. This is because the duration of four oscillations with a frequency of 200 Hz (wavelength 5 ms) is 20 ms.

**FIGURE 1:**
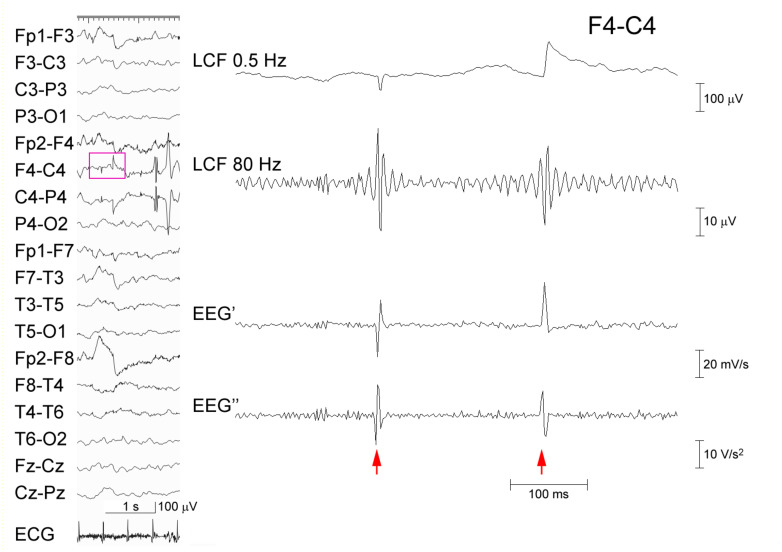
Processing of EEG artifacts. Two artifacts that were caused by poor electrode contact at C4 (left panel). The EEG data at F4–C4 (pink rectangle) that were recorded from a 5-year-old boy is temporally expanded (right panel) (abrupt potential jumps at arrows). From the top: EEG processed with an FIR low-cut frequency (LCF) filter at 0.5 Hz; EEG LCF filtered at 80 Hz showing spurious oscillations; EEG’; and EEG″ showing no ringing. The corresponding time–frequency analysis is shown in Supplementary Figure 1A.

**FIGURE 2:**
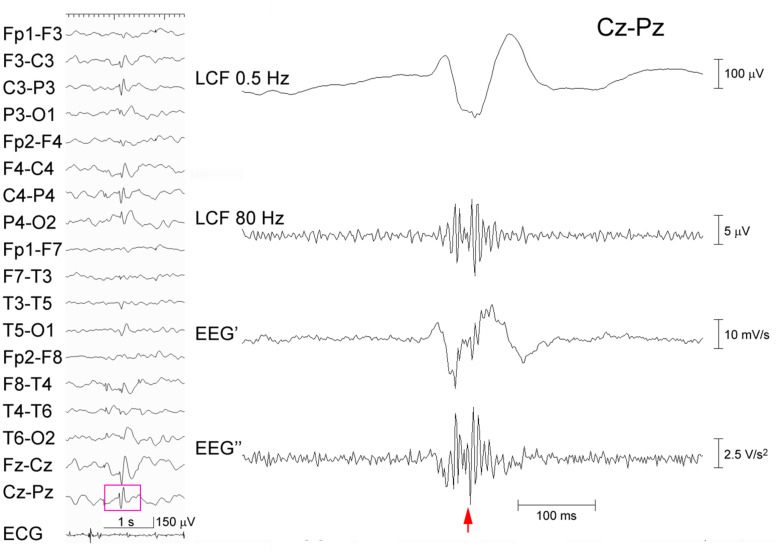
Processing of an EEG epileptic discharge. A spike-wave recorded from a 5-year-old boy (left panel). The EEG data (pink rectangle) at Cz–Pz including the spike (arrow) is temporally expanded and processed (right panel) as in Figure 1. Ripple oscillations can be seen in both the trace LCF filtered at 80 Hz and the EEG″ trace. The corresponding time–frequency analysis is shown in Supplementary Figure 1B.

Figure 1 shows a representative example of ringing at the arrows that is caused by a type of FIR filter based on application of the discrete Fourier transform, zeroing parameter values below 80 Hz, and the subsequent inverse Fourier transform. Note that the EEG’ and EEG″ traces do not cause ringing. Conversely, Figure 2 shows a spike that was recorded from a representative pediatric patient with focal epilepsy and was processed similarly, as follows: ripple oscillations are clearly observed in the FIR filtered trace and the EEG″ trace. Therefore, it is evident that ripples can be found in at least some EEG″ traces without concerns about the possibility of false ripples. However, it is still unknown how efficient it is to detect ripples particularly from routine, possibly noisy, pediatric EEG records. The present study was designed to clarify this issue.

### Subjects

Fifty pediatric patients who visited Okayama University Hospital from January 2017 to August 2019 and who were 3–13 years old at the time of the scalp EEG recording that showed frequent spikes with a presumed focal origin during sleep were enrolled into the study. The participants were limited to those who had not yet undergone an EEG analysis. Children with such an EEG abnormality pattern were selected because they tend to show ripples in association with spikes (Ohuchi et al., 2019). The demographic data of these patients are shown in Table 1.

**TABLE 1:** Demographic data of the patients.

Age		7.93 ± 2.53 years				
		Number of pts.	Number of spikes with ripples	Mean peak frequency of ripple-blobs (Hz)	Mean peak power of ripple-blobs (μV^2^)	Mean duration of ripples (ms)
Total		50 [14]	57 (1.14)	114.4 ± 18.6 {80.1–160.2}	0.46 ± 0.62 {0.05–3.77}	98.8 ± 28.6 {52–186}
**Sex**						
	Male	32 [7]	22 (0.69)	110.2 ± 17.1 {82.0–140.6}	0.29 ± 0.21 {0.10–0.78}	97.8 ± 34.5 {52–186}
	Female	18 [7]	35 (1.94)	117.1 ± 19.2 {80.1–160.2}	0.56 ± 0.76 {0.05–3.77}	99.4 ± 24.7 {58–146}
**Disorder**						
	Childhood epilepsy with centrotemporal spikes	6 [2]	2 (0.33)	98.7 {87.9, 109.4}	0.19 {0.13, 0.25}	96 {88, 104}
	Panayiotopoulos syndrome	3 [0]	0 (0)	N/A	N/A	N/A
	Epilepsy with coexisting generalized and focal abnormalities	2 [0]	0 (0)	N/A	N/A	N/A
	Focal epilepsy evolving to epileptic encephalopathy with continuous spike-and-wave during sleep or related disorder	8 [7]	41 (5.13)	117 ± 18.8 {80.1–160.2}	0.50 ± 0.71 {0.05–3.77}	95.5 ± 28.5 {52–186}
	Other focal epilepsies with various etiologies	21 [5]	14 (0.67)	109.2 ± 17.4 {82–140.6}	0.37 ± 0.27 {0.12–1.02}	109 ± 29.4 {64–172}
	EEG spikes without clinical seizures	10 [0]	0 (0)	N/A	N/A	N/A

*pt(s), patient(s); (), group mean; [], pts having at least one spike with ripples; {}, range; N/A, not applicable; ±, mean and standard deviation.*

This study was approved by the Okayama University Ethics Committee (approval No. 1911-024).

### Methods of Analysis

Electroencephalography data were recorded with a sampling rate at 500 Hz using a Nihon-Kohden (Tokyo, Japan) Neurofax system, which used a low-cut frequency filter at 0.08 Hz before digital sampling. The international 10–20 electrode system was used. Computation was performed using a program written in-house for MATLAB (version 7.5.0; MathWorks Inc., Natick, MA, United States).

For each participant, ten consecutive spikes were selected with a minimal interval of 1.5 s. Five hundred data segments with a duration of 1.4 s, among which each segment including a spike at the mid-point, were initially analyzed in a single bipolar channel that showed clear spike morphology; these spike-data were processed using a combination of TFA and an FIR low-cut filter at 80 Hz. In each spike-data segment, after discarding the beginning and ending 400-ms part, the middle 600-ms part was reviewed by two experienced electroencephalographers using consensus to identify the presence or absence of ripples that had ≥4 oscillations and an amplitude that was clearly above the background signal according to Andrade-Valenca et al. (2011). In this study, the ripple-associated spectral blobs should have peak power ≥0.05 μV^2^ in TFA (Session A).

These EEG data segments were subjected to numerical differentiation as explained above. The EEG″ trace of the middle part of each spike-data segment was presented to two other electroencephalographers without information about the results in Session A. The order of presentation was randomized. The latter electroencephalographers reviewed each EEG″ trace independently to judge for the presence of clear ripple oscillations, uncertain oscillations, or the absence of oscillations (Session B).

### Statistical Analysis

In the judgments in Session B, the strict criterion for ripples was defined to include only clear ripples, and the extended criterion included clear and/or uncertain ripples. The judgments in Session B were compared to the initial categorization in Session A using Fisher’s exact test.

## Results

In Session A, ripples were identified in 57 spike-data segments (Group A-R) that were recorded from 14 children and not in the other 443 segments (Group A-N), as shown in Table 1. Ripples were predominantly observed in patients with focal epilepsy that evolved to epileptic encephalopathy with continuous spike-and-wave during sleep or related disorders. Conversely, ripples were rare and variable in focal epilepsies, and lacking in patients with spikes with no clinical seizures.

In Session B, as indicated in Table 2, both reviewers consistently found clear ripples (strict criterion) in the 11 spike-data segments, which were all included in Group A-R and not in Group A-N (*p* < 0.0001; sensitivity 19.3%, specificity 100%). For the inter-rater agreement between the two reviewers in Session B, the Kappa coefficient was 0.331.

**TABLE 2:** Identification of ripples in Sessions A and B.

			Session A: ripples identified by a combination of filtered EEG and time-frequency analysis		Sum	Statistics (Fisher’s exact test)
			Present (Group A-R)	Absent (Group A-N)		
Session B: ripples identified in EEG″ traces	Strict criterion (clear ripples alone)	Detected	11	0	11	*p* < 0.0001
		Not detected	46	443	489	
	Extended criterion (clear and/or uncertain ripples)	Detected	23	1	24	*p* < 0.0001
		Not detected	34	442	476	
Sum			57	443	500	

**FIGURE 3:**
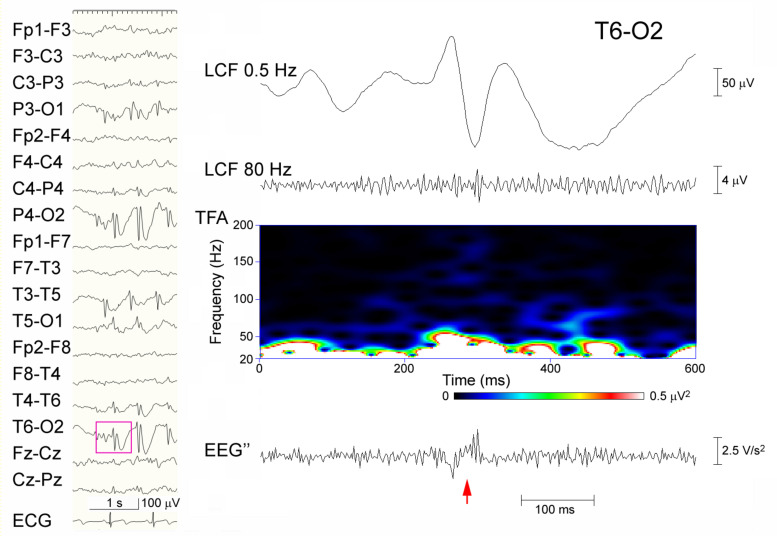
Discordant judgment in an epileptic discharge (possible ripples that were not identified in Session A but identified using the extended criterion in Session B). Spike-waves are dominant over the right occipital region that was recorded from an 8-year-old girl (left panel), and the part of EEG data (pink rectangle) at T6–O2 including the spike (arrow) is temporally expanded and processed (right panel). From the top: filtered at 0.5 Hz; filtered at 80 Hz with background oscillations; time–frequency analysis (TFA) lacking clear spectral blobs; and the EEG″ trace showing spike-associated oscillations surrounded by noise-like background activity. In Session A, this spike was categorized as devoid of ripples, whereas in Session B, it was judged to include clear ripple oscillations by one reviewer and to include uncertain oscillations by the other.

**FIGURE 4:**
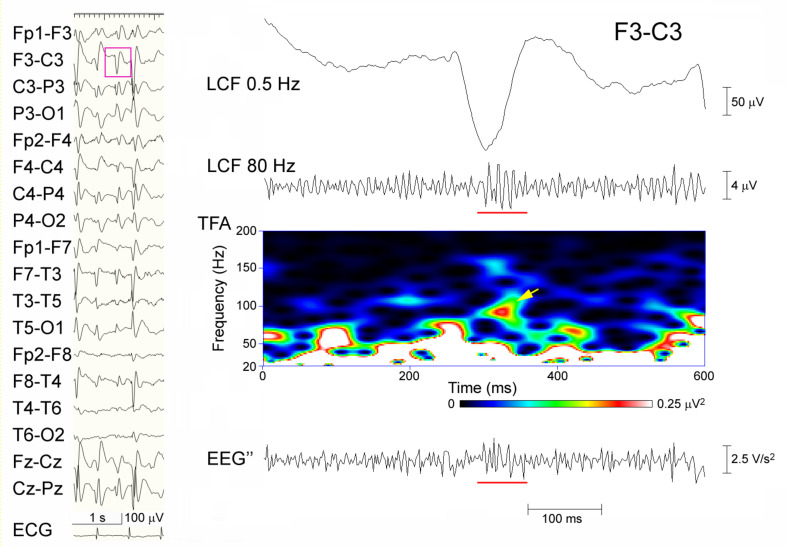
A representative failure to detect ripples in Session B. Spike-waves are almost generalized in a sleep EEG that was recorded from an 8-year-old girl (left panel), and the EEG data (pink rectangle) at F3–C3 including the spike is temporally expanded and processed (right panel). From the top: filtered at 0.5 Hz; filtered at 80 Hz with ripple oscillations (underline) and background noise; TFA showing a clear spectral blob (arrow); and the EEG″ trace showing some spike-associated oscillations (underline) buried in noise-like background activity. In Session A, this spike was categorized as having ripples, whereas in Session B, neither of the reviewers judged it to include ripple oscillations.

When the extended criterion of ripples was used in Session B, there were 25 spike-data segments, which fulfilled the criterion and was agreed-upon by the two reviewers; 24 of these were in Group A-R and the remaining one was in the Group A-N (*p* < 0.0001; sensitivity 42.1%, specificity 99.8%; Kappa coefficient of the inter-rater agreement 0.391). There was only one spike-segment that was categorized as Group A-N for Session A and fulfilled the extended criterion in Session B (judged to show clear ripples by one reviewer and to show uncertain ripples by the other reviewer). In this segment, there were considerable background oscillations in the initial FIR filtered EEG in Session A, and the background activity looked irregular and noise-like on the EEG″ trace, showing discernible ripples in Session B (Figure 3). Spike-data surrounded by heavy background noise signals tended to result in failure of ripple identification in Session B (Figure 4).

## Discussion

We have demonstrated that real ripples over scalp spikes exist in some patients, and that these real ripples can be distinguished from false ripples using numerical differentiation of EEG data. The possibility of false ripples might make researchers uneasy, but the present findings will form the basis to study scalp HFOs particularly in children with developmental and epileptic encephalopathy. There should be true ripples that have a relatively low amplitude in a noisy background, and thus, cannot be clearly depicted in the EEG″ traces. Ripples that are visualized consistently using both ordinary filters and the EEG″ method should be true, but failure to clarify the ripples using the EEG″ method does not mean that the true ripples are not present.

Although the combination of TFA and EEG filtering that was used in Session A is a standard method to identify HFOs, true HFOs that are buried in slower potentials may not always build isolated peaks, and filtration may generate redundant oscillations at spikes and in the background. As illustrated in Figure 3, the spike that showed ripples based on the extended criterion in Session B and no ripples in Session A may include some sort of true oscillations that were not easily discernible from the noisy background in the ordinary-filtered EEG. The EEG″ traces that were shown to have a high specificity may supplement these methods to recognize true ripples. The modest sensitivity of the EEG″ technique may be due to the effects of confounding background noise, as shown in Figure 4. There is no single perfect analysis method, and we do not have the ground truth or ideal baseline data to assess the true performance of each available method. We hope that future studies using simulated data will help to solve these issues.

The 500 spikes that were used in the present study were not intentionally selected using previous information on ripples and the background noise level. Spikes in epileptic encephalopathy with continuous spike-and-wave during sleep and related disorders tended to have associated ripples in the present study, but ripple-laden spikes were rare and variable in children with other types of disorders, as already reported (Ohuchi et al., 2019). The presence of only 57 spikes with ripples in Group A-R suggests that ripples are generally rare events that should have a special meaning.

The EEG’ and EEG″ traces can be regarded as FIR-filtered EEG data, but they have particular meanings. EEG data are summed potentials that are generated by many neurons. The EEG’ traces that show the degree of instantaneous change in the EEG may represent the driving forces behind the EEG changes, which may reflect the net amount of synaptic input at the moment more so than the raw EEG data. The EEG″ traces show the tendency for changes in the EEG’ or possibly the direction of EEG changes that are taking place. This type of viewpoint for EEG is expected to increase the utility of EEG.

There are limitations and unsolved questions in the present study. The number of spike samples, particularly spikes with ripples, was not large, and we need to involve much more spike- and non-spike-data to clarify the effects of the EEG″ method. We used a Δ*t* of 2 ms because the sampling rate was 500 Hz, but the selection of Δ*t* may influence the results. We have not yet determined the best Δ*t* for EEG″. Additionally, we have not compared the effects of various options for the numerical approximation of the second derivative. We should address these questions in the future.

The inter-rater agreement in Session B was modest, suggesting that experience is needed to share the common knowledge and observe EEG″ traces. The EEG″ method was new to the reviewers, and establishment of a consensus standard regarding its interpretation is another future issue. This is why judgment was not based on consensus in Session B, which is in contrast to Session A that involved the already established analysis method, i.e., the combination of TFA and an FIR. When reviewers are accustomed to the EEG″ method, the quality of identification may improve.

Roehri et al. (2016) investigated several methods of whitening, including the first derivative, but not the second derivative, to improve the detectability of HFOs. The present EEG″ method is very simple, and there might be more sophisticated methods that have better performance in detecting HFOs compared to EEG″. Excluding the possibility of “false ripples,” however, has not been rigorously pursued to the best of our knowledge. If this EEG″ method is used as an adjunctive tool in addition to the information about ordinary filtered or whitened EEG, then the preciseness with which the ripples may be identified will be improved. Despite all these limitations, we hope to add the numerical differentiation of EEG as another way to review EEGs that include spike-associated ripples and other types of high frequencies. Because there are pitfalls of bias in the detection of scalp HFOs (Gerner et al., 2020), we would like to refine the EEG differentiation method to develop a methodology to avoid such pitfalls and to make scalp HFOs a truly useful biomarker in the future.

## Conclusion

Numerical differentiation of EEG data would provide proof for the presence of such unusually fast oscillations or ripples over the scalp and support the importance of this electrophysiological phenomenon.

## Data Availability Statement

The raw data supporting the conclusions of this article will be made available by the authors, without undue reservation.

## Ethics Statement

The studies involving human participants were reviewed and approved by the Okayama University Ethics Committee (approval No. 1911-024). Written informed consent from the participants’ legal guardian/next of kin was not required to participate in this study in accordance with the national legislation and the institutional requirements.

## Author Contributions

KK designed the methodology, conducted the analysis, and wrote the manuscript. KK and TA served as EEG reviewers in Session A. TS and HT reviewed the data in Session B. All authors contributed to the article and approved the submitted version.

## Conflict of Interest

The authors declare that the research was conducted in the absence of any commercial or financial relationships that could be construed as a potential conflict of interest.

## Supplementary Material

Supplementary Figure 1 Time–frequency analysis of sample EEG data. **(A)** A spectral panel including two artifacts (arrowhead and arrow) that were produced from the EEG data at F4–C4, which is shown in Figure 1. **(B)** Spectral data including a spike with ripples (arrow) that were produced from the EEG data at Cz–Pz, which is shown in Figure 2.
