# Supplementary figures and images for: Exclusion of the Possibility of “False Ripples” From Ripple Band High-Frequency Oscillations Recorded From Scalp Electroencephalogram in Children With Epilepsy
Source: Front Hum Neurosci. 2021 Jun 15;15:696882. doi: 10.3389/fnhum.2021.696882 (PMC8239160; doi:10.3389/fnhum.2021.696882)

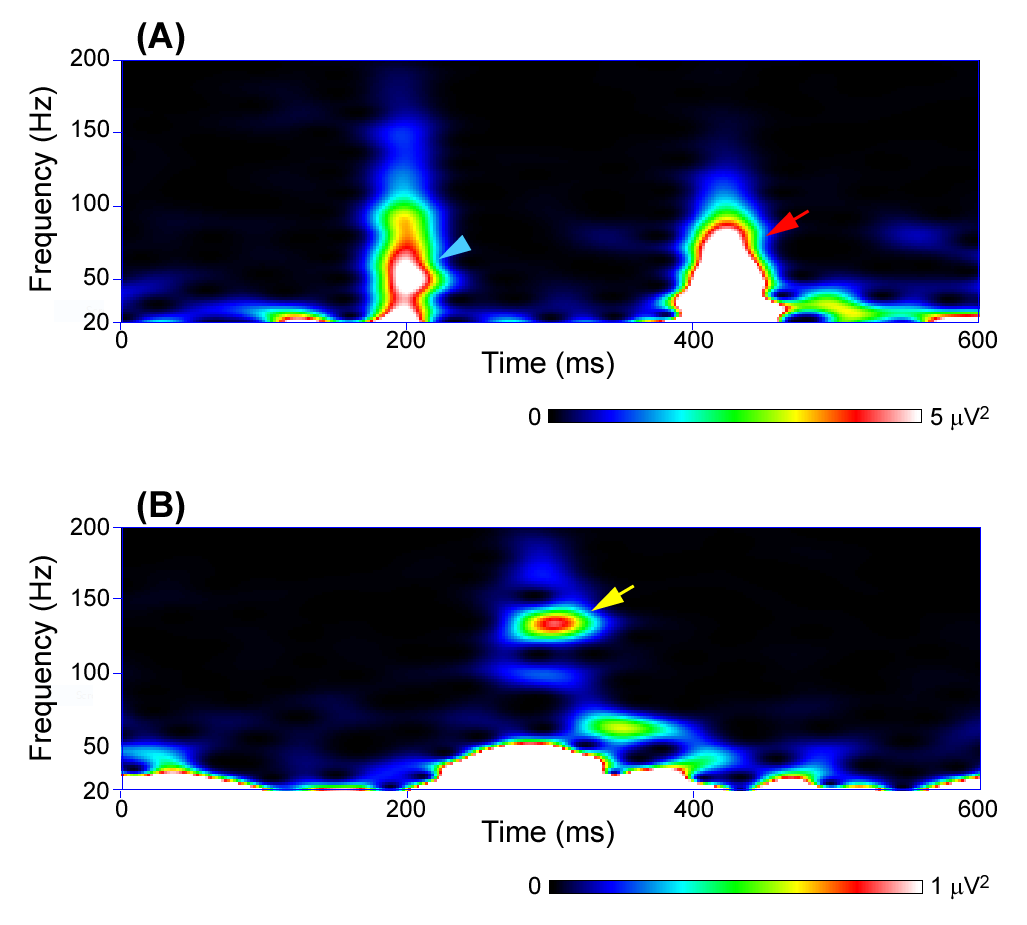

Supplement: Supplementary Figure 1 — Time–frequency analysis of sample EEG data. (A) A spectral panel including two artifacts (arrowhead and arrow) that were produced from the EEG data at F4–C4, which is shown in Figure 1. (B) Spectral data including a spike with ripples (arrow) that were produced from the EEG data at Cz–Pz, which is shown in Figure 2. [file Image_1.TIF]
